# The application of the spot the difference teaching method in clinical skills training for residents

**DOI:** 10.1186/s12909-022-03612-3

**Published:** 2022-07-14

**Authors:** Liu Yang, Wen Li, Jian Zou, Junnan An, Bin Zeng, Yitao Zheng, Jiming Yang, Jia Ren

**Affiliations:** Department of Otolaryngology Head & Neck Surgery, West China Hospital, Sichuan University, 37 E Guoxue Alley, Chengdu, Sichuan 610041 People’s Republic of China

**Keywords:** Clinical education, Game-based learning, Observation learning, Flipped learning, Peer-assisted learning, History-taking training, Basic surgical skills training

## Abstract

**Background:**

Clinical skill training (CST) is indispensable for first-year surgical residents. It can usually be carried out through video-based flipped learning (FL) within a web-based learning environment. However, we found that residents lack the process of reflection, blindly imitating results in losing interest and passion for learning in the traditional teaching pattern. The teaching method of "spot the difference" (SDTM), which is based on the fundamentals of the popular game of "spot the difference," is designed to improve students' participation and reflective learning during skill training. This study aimed to evaluate this novel educational model's short-term and long-term effectiveness for surgical residents in China.

**Methods:**

First-year residents who required a three-month rotation in the head and neck surgery department were recruited to participate in a series of CSTs. They were randomized into SDTM and traditional FL (control) groups. Clinical skill performance was assessed with validated clinical skill scoring criteria. Evaluations were conducted by comparing the scores that contain departmental rotation skill examinations and the first China medical licensing examination (CMLE) performance on practical skills. In addition, two-way subjective evaluations were also implemented as a reference for the training results. Training effects were assessed using t tests, Mann–Whitney–Wilcoxon tests, chi-square tests, and Cohen’ s effect size (d). The Cohen’ s d value was considered to be small (<0.2), medium (0.2-0.8), or large (>0.8).

**Results:**

The SDTM group was significantly superior to the control group in terms of after-department skill examination (t=2.179, *p*<0.05, d=0.5), taking medical history (t=2.665, *p*<0.05, d=0.59), and CMLE performance on practical skill (t=2.103, *p*<0.05, d=0.47). The SDTM members rated the curriculum more highly than the control on the items relating to interestingness and participation (*p* < 0.05) with large effect sizes (d >0.8). There were no significant differences between the two groups on clinical competence (t=0.819, *p*=0.415, d=0.18), the first-time pass rate for CMLE (χ2 =1.663, *p*=0.197, d=0.29), and short-term operational skills improvement (t=1.747, *p*=0.084, d=0.39).

**Conclusions:**

SDTM may be an effective method for enhancing residents' clinical skills, and the effect is significant both short- and long-term. The improvement effect seemed to be more significant in the peer-involved SDTM than training alone. However, despite positive objective results, SDTM still risks student learning burnout.

**Trial registration:**

ISRCTN registry, ISRCTN10598469, 02/04/2022，retrospectively registered.

**Supplementary Information:**

The online version contains supplementary material available at 10.1186/s12909-022-03612-3.

## Background

Patient safety is an ongoing subject in the medical field, and it is also the most basic starting point and the ultimate goal of medical services. Against this background, the clinical skills training (CST) mode has transferred from patient-oriented to video-based and simulation-based flipped learning (FL )[1, 2]. This traditional pedagogical method consists of two steps: watching the standard and flawless guide videos before class; implementing the practical operation, and assessing learning effectiveness in class. Although this teaching approach enables junior residents to avoid the risks associated with training directly in the clinical setting, the lack of introspection, dwindling interest in learning, and losing realistic sensory stimuli limits the training effectiveness. The development and implementation of flipped classroom teaching on CST still has some challenges, including preparing and improving preclass materials, active participation, and persistent innovation. This finding gives us some implications that medical education researchers should explore new points that can be combined with FL to extend the efficiency of clinical skills training [3].

Spot the difference is a popular childhood game associated with a prototypical change blindness task and involves the identification of differences in local features of two otherwise identical scenes using an eye scanning and matching strategy [4]. During the game, the right posterior parietal cortex (RPPC) and visual centers are doubly activated. The connections between the two regions of the cerebral cortex can be built and affected [5]. The RPPC is concerned with the imitation of the actions of others, and the region displays a specific function in processing spatial aspects of complex movements [6, 7]. Imitation is the first and most significant step for clinical skill acquisition and is associated with precision and complex actions, so the instructional approach associated with the spot the difference game might be effective for clinical skills improvement. In addition, stimulating the RPPC could also promote the encoding of long-term memories that provides a possibility to develop a novel teaching method for achieving long-term learning effects [8]. Moreover, the RPPC played an essential role in the guidance of attention, which laid a theoretical foundation for our consideration of cultivating concentration during learning [9].

The spot the difference teaching method (SDTM) is based on this game. We aim to apply a similar version of the game to CST to stimulate students' enthusiasm and participation that results in satisfactory learning effects. The commonalities between SDTM and the method of problem-based learning (PBL) are problem detection by learners, teacher guidance, problem discussion, and conclusion [10]. PBL has situation dependencies, while SDTM does not. Because SDTM's questions are designed by the teacher before class, the correct answers are constant. Thus, the latter is more flexible and its operation is easy for teachers to accept. In a broad sense, the differences are no longer limited to faults or problems, or they could be outstanding merits. SDTM does not follow the rules of the game completely. An improved teaching method based on game theory is observing the differences or faults surrounding a movement to determine how to perform the skill through self-reflection. SDTM is a novel teaching model integrating observation learning, PBL, guided learning, and student-teachers cooperative learning or peer-assisted teaching. We hypothesized that applying the SDTM to CST might enhance the efficiency of education based on the RPPC play in cognitive functions of learning and memory.

Taking medical history is not only the first step for doctors to diagnose and treat patients but also provides a critical opportunity for doctor-patient communication and the establishment of a sound doctor-patient relationship. However, clinical training often fails to equip medical students with essential history-taking skills [11]. History-taking skill training, which is considered a cultivating clinical reasoning ability, is indispensable for first-year medicine residents. Practicing history-taking depends on standardized patient training and constant improvement of a simulated patient based on artificial intelligence [12]. However, the lack of student engagement and insufficiency of student supervision limit the history-taking training effect, because the course lacks teacher-student and peer interaction within a noncommunicative environment [13]. We hope the situation will improve through the well-designed SDTM.

Cardiopulmonary resuscitation and basic operations related to surgery are the skill contents that must be mastered for first-year surgical residents. These skills are the essential test points of the China medical licensing examination (CMLE) scheduled for the second year of employment. These basic skills must be mastered before attempting more complex tasks, and bad habits learned early are difficult to correct [14]. However, temporal and spatial constraints loom large during the training process [15]. The SDTM might provide a refined perspective on the efficiency of the skill education program. In addition to cardiopulmonary resuscitation, we selected three basic surgical operations as the training and assessment contents, including dressing change, disinfection and surgical drape placement in the operation area, wearing and taking off the operating gown, and sterile gloves.

To verify SDTM’s validity in improving the effectiveness of clinical skills training, we selected first-year residents with the same baseline on the entrance examination to participate. They were randomized into experimental and control groups that differed concerning the teaching methods: SDTM was adopted in the experimental group, while the traditional teaching method was adopted in the control group. We considered the departmental rotation examination (DTE) results and the two-way subjective evaluation between students and teachers as the short-term training effect. We regarded the performance of first-time CMLE as the long-term teaching effect. We statistically compared the short-term and long-term results between the two groups. We assumed that the application of SDTM would result in superior skill learning results compared with the control group.

## Methods

### Study design and participants

**Fig. 1:**
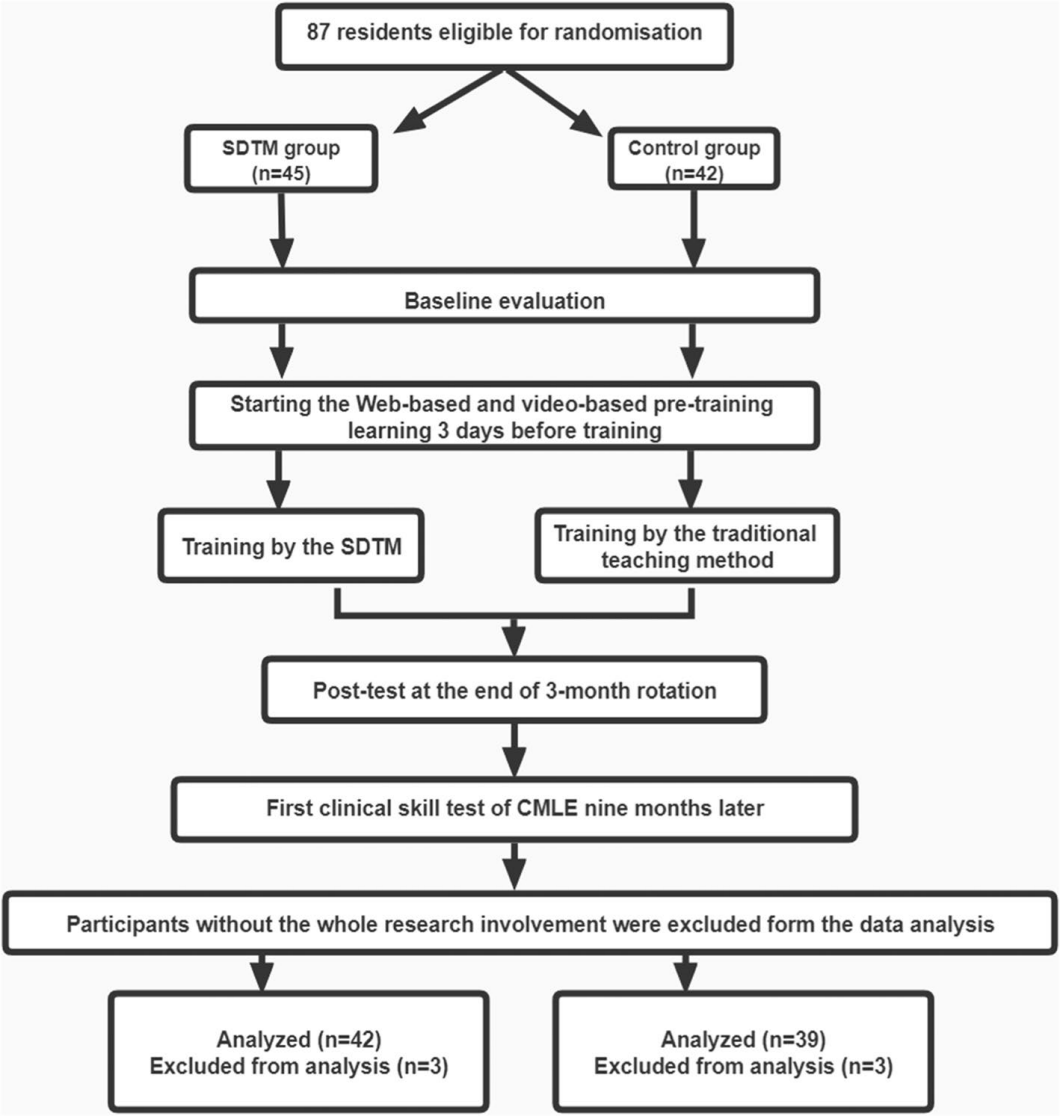
Depicts the research flow and procedures

A summary of the study profile is shown in Fig. 1. We enrolled first-year residents who required a three-month rotation in the Department of Head and Neck Surgery at our hospital from September 2019 to September 2020. The inclusion criteria were as follows: 1) first-year residents who did not rotate to other surgical departments and 2) those who obtained a bachelor's degree in medicine. The exclusion criteria were as follows: 1) residents who had rotated to other surgical departments before the study; 2) senior residents (working time ≥ 1 year); 3) residents with a graduate degree; 4) residents who obtained the qualification certificate of a clinical practitioner. After screening, a total of 87 residents were selected as the research objects. They were divided into eight subgroups in the order of rotation, and the subgroups were randomly sorted into either a traditional teaching group (the control) (n =42) or an SDTM group (n =45) using a web-based randomization program. All of them had passed the entrance test, which was used as the baseline evaluation, and they would take the CMLE 9 months after the end of the rotation. Participation was voluntary, and informed consent was obtained. The students did not know which group they were randomized to, and the teacher grading the students did not know which group he or she belonged to. Participants were assured that all data would be treated anonymously. Each subgroup contained an SDTM and the control group, and there were three to six members in each of the two groups. Each subgroup was supervised by the tutorial group consisting of two instructors who held full-time professional positions within the head and neck surgery department and three assistants. The teachers grading the residents included two professionals (who graded the exam objectively) and four clinical teachers (who graded the clinical performance subjectively). They did not participate in the teaching process and had a professional title above the senior associate position. They were all practicing clinical physicians. The study was approved by the Institutional Review Board and Ethics Committee of the West China Hospital of Sichuan University.

### Preparation phase

#### History-taking training preparation

First, four scripts were written for a standardized patient composed of two instructors with SP training certificates. Second, a standard medical history-taking video (approximately 15 minutes) was recorded based on acute suppurative tonsillitis, which did not belong to the above scripts. Third, an email containing the above recorded video and a PDF of history-taking scoring standards was sent to all the participants three days ahead of the training course. The SPs were familiar with the scripts and rehearsed at least once with a senior resident before class. Both the SDTM and the control group were asked to watch the video at least once and review the PDF file to understand the critical point of history-taking.

#### Other introductory clinical skills training preparation

First, the instructor prepared flawless CST videos (approximately 8 minutes) about cardiopulmonary resuscitation, debridement and dressing change, disinfection, placing surgical drapes in the operation area, wearing and taking off the operating gown, and wearing sterile gloves. These videos were implemented according to CMLE guidelines. Second, this is a critical point of this study called "the difference points design." The difference points (DPs) were error-prone points that are often overlooked in daily clinical skills operations. The DPs were discussed and decided by the teaching group members. Approximately three DPs were arranged to be displayed in each standard video (Table 1). These videos containing the DPs were one-to-one correspondence with the standard videos, and the same performers recorded the flawed videos in the same settings. Finally, three days before class, the residents in the same subgroup received an email including a PDF of the point checklist on skill operation and the flawless videos. Every standard video was sent in an orderly manner according to the curriculum schedule. Both groups needed to observe the video at least once and review the critical point checklist of each operation.

**Table 1:** Difference points

Standard operation procedure	Difference points
**Cardiopulmonary resuscitation**	
Correct compression depth (1.5–2 inches) and fingers off the chest	Compression depth (approximately 0.5 inches) and fingers on the chest
Remove secretions and foreign bodies from the mouth and nose and keep the airway opening (head tilt and chin lift)	Forget to remove secretions and foreign bodies of the airway and the chin is not lifted before the artificial respiration
Judge the resuscitation effect after five cycles	Judge the resuscitation effect after six cycles
**Dressing change**	
Remove the inner dressing with a tweezers	Remove the outer and inner dressing with the hand
The second disinfection scope should be narrower than that of the first	The second disinfection scope is equal to that of the first
A pair of tweezers contacts the incision, and the other is used to transfer the cleaning items of the dressing bowl.	Two pairs of tweezers are mixed-use
**Wearing and taking off the operating gown and sterile gloves**	
Extend the hands forward (not over the shoulders) during the dressing process	Extend the hands upward slightly (over the shoulders) during the dressing process
The gloved hand shall not touch the inner surface of the other glove	The thumb of the gloved hand touches the inner surface of the other glove
With the help of an assistant, take off the operating gown first and then the gloves	Take off the gloves first by oneself and then the operating gown with the help of an assistant
**Disinfection and surgical drapes placement (neck)**	
Disinfection range: Up to the lower lip, down to the nipple line, both sides to the anterior edge of the trapezius muscle	Disinfection range: Up to the submental plane, down to the clavicular pane, both sides to the anterior edge of the sternocleidomastoid
Keep the tip of the sterilizing forceps lower than the holding end	The tip of the sterilizing forceps is occasionally over the handheld end
Place four towels in the correct order: the lower part, opposite side, head side and trainee side	Place four towels in the wrong order: opposite side, lower part, trainee side and head side
**Medical history-taking**^a^	
Remember to ask about the predisposing factors	Forget to ask about the predisposing factors
Ask about the negative symptoms in detail	Ignore some negative symptoms inquiry
Good professional quality	Poor professional quality

^a^Since the DPs of medical history-taking are not aimed at a certain disease, the DPs are for reference only as examples.

### Curriculum arrangement

Since rotation began, CST courses were arranged every two weeks. The length of each class varied slightly depending on the number of students, averaging approximately 120 minutes per group. The control group and experimental group were placed in separate rooms because all participants were asked to be discreet about the details of their training and not to disclose them to their fellow residents. The curriculum was arranged in the following order: history-taking, cardiopulmonary resuscitation, dressing change, disinfection and surgical drape placement in the operation area, wearing and taking off the operating gown and sterile gloves.

### History-taking training program

The SDTM group members were randomly ranked, and a printed scoring sheet whose contents were the same as the previous PDF was distributed to everyone before class activities began. Then, the resident orderly performed a history-taking process of the standard patient. Each resident performed a script that was selected from the four prepared scripts. The remaining members were asked to watch the process and mark the deficiencies on the scoring sheet when they thought they spotted the difference from the standard procedure. There are some examples of DPs related to medical history-taking shown in Table 1. In addition, if they observed some points that the testee performed better than the standard answers, they could also write them down. Each resident would receive all the feedback items from other members at the end of the class, and the instructor summarized the class and went over the frequent errors raised during this course.

The preparation of the control group before class was the same as that of the experimental group while they were taught traditionally. Each trainee was trained individually. This training took place in a quiet room with only the trainee, the SP, and the instructor. The instructor scored their performance, pointed out frequent weaknesses, and put forward some suggestions for improvement on the spot.

### The introductory clinical skills training program

A manikin was used for clinical skill training in both groups. Before their clinical skills training, the SDTM group watched the video containing the DPs, while the control group reviewed the previous standard video. Both the control group and the experimental group were given one-on-one training in random order. The SDTM group members were required to promptly mark the flawed point on the scoring sheet when they spotted the difference from the standard procedure. After that, every resident performed the skill operation individually, and the trainee was asked to tell loud how to operate the modified skill when they got to a different point. If some differences were not detected or other operating defects existed, the instructor would tell the corresponding participant at the end of class.

In the control group, the instructor organized the skill training following the previously recorded standard video guidance and provided individualized feedback at the end of training.

### Outcome measure and statistical analysis

The two-way subjective evaluation between students and teachers and DTE evaluated the short-term teaching effect. The DTE was the test at the end of the rotation internship, and it contained a history-taking test and a basic clinical skill test. The script for the history-taking test was randomly selected from the other three scripts, and it was different from the training script. The checklist for history-taking skill evaluation (a maximum of 15 points) is shown in Additional file 1. Only one introductory clinical operating skill could be randomly selected for evaluation due to time limitations. Both groups were assessed with the items of the checklists (a maximum of 100 points) displayed in Additional file 2 (including four sheets). All the participants were evaluated one by one in random order by one blinded reviewer, and all the scoring criteria standards were formulated following the 2020 CMLE. The students' subjective questionnaire was designed based on a 5-point Likert scale, and it included five items (5 = agree, 1 = disagree). The items of the questionnaire were chosen according to a previous report and our teaching experience [16]. Each resident anonymously assessed the quality of teaching received using the questionnaire after the rotation. Each student's clinical teacher would score his or her daily clinical performance subjectively after rotation immediately, and the evaluation involves the following three aspects: theoretical knowledge, independent learning capability, and doctor–patient communication skills.

The long-term teaching effect was evaluated by the clinical skill performance of first-time CMLE, which was considered a 9-month retention test used to assess each individual's skills. The aggregate scores of the test and the pass rate were used as the evaluation indicators.

We compiled the total score for each test. When a normal distribution was present, the results generated by each of the two groups were compared using an independent sample T test and reported as the mean±SD. Otherwise, nonparametric test methods, such as the Mann–Whitney–Wilcoxon test, were used, and the result could be reported as a median (lower quartile, upper quartile). The chi-squared test was used to analyze the difference in the rate between the two groups. All statistical analyses were performed using SPSS software (version 22.0). P values of less than 0.05 were considered significant. Cohen’ s d effect size was calculated to identify the magnitude of any differences between the both groups. The practical effect size proposed by the Cohen’ s d value was considered to be small (<0.2), medium (0.2-0.8), or large (>0.8, [17].

## Results

### Demographic data and baseline information

A total of 87 residents were invited to participate in this study according to the research criteria. They all passed the entrance examination (theory 70% and skill 20%) before the rotation. There were 42 participants in the SDTM group, and 39 in the control group were included in the final analysis. The test results of the two groups showed no significant difference, which confirmed that both groups had the same baseline. There were no significant differences between the two groups regarding gender and age (P > 0.05) with small effect sizes (Cohen’s effect size (d)< 0.2). Three participants in the experimental group and three in the control group dropped out of the research during the process due to unavoidable causes. Because data were analyzed continuously with correlative measures, 6 of 87 residents were excluded from the final analysis. The demographic and baseline data are shown in Table 2**.**

**Table 2:** The demographic and baseline data

	Control group	SDTM group	Statistics	*P*-value	d^a^
Total number of residents	39	42			
Age (years, mean ± SD)	23.77 ± 1.062	23.81 ± 1.041	T = 0.172	0.864	0.04
Sex (male/female)	19/20	22/20	χ2 =0.109	0.742	0.07
Baseline score (max 100 points, mean ± SD)	87.38 ± 3.368	87.26 ± 3.147	T = 0.169	0.866	0.04

^a^Cohen’s effect size (d)

### Evaluation of short-term teaching effect

#### Objective parameters comparison

The performances of the departmental rotation examination are shown in Table 3. The total SDTM score was significantly higher than that of the control (*P* <0.005) with a medium effect size (d = 0.50). In the SDTM group, the mean history-taking score and the introductory clinical operating skill score were 10.19 ± 1.348 and 87.40 ± 3.895, respectively. Meanwhile, for the traditional group, they were 9.44 ± 1.188 and 85.69 ± 4.899, respectively. There was a significant difference in history-taking performance scores between the two groups (*P* <0.001) with a medium effect size (d = 0.59). Although the SDTM group’s score was higher than that of the control in the short-term results of operating skill training (87.40 versus 85.69), there was no statistically significant difference (*P* =0.084) with a medium effect size (d = 0.39).

**Table 3:** Objective parameter comparison of the short-term teaching effect

	Control group	SDTM group	Statistics	*P* value	d^a^
Total score of departmental rotation examination (max 115 points)	95.13 ± 5.424	97.60 ± 4.762	T = 2.179	0.032	0.50
History-taking score (max 15 points)	9.44 ± 1.188	10.19 ± 1.348	T = 2.665	0.009	0.59
Operating skill score (max 100 points)	85.69 ± 4.899	87.40 ± 3.895	T =1.747	0.084	0.39

^a^ Cohen’s effect size (d)

#### Two-way subjective evaluation between students and teachers

As shown in Table 4**,** there was no significant difference in the total scores from clinical teachers’ subjective evaluation between the SDTM group and the control group (*P* = 0.415, d=0.18) or the level of mastery of theoretical knowledge. However, the comparison of independent learning capability showed that the control group’s performance was better than that of the SDTM group (*P* < 0.005) with a medium effect size (d= 0.53). Notably, the SDTM group’s doctor–patient communication capability received a higher rating than the control group’s (*P* < 0.005) with a large effect size (d = 0.90).

**Table 4:** Scores from the subjective evaluation of clinical teachers

	Control group	SDTM group	Statistics	*P* value	d^a^
Total score (max 100 points)	90.00 ± 2.883	90.59 ± 3.589	T =0.819	0.415	0.18
Theoretical knowledge (max 40 points)	35.20 ± 1.908	35.50 ± 2.319	T = 2.665	0.536	0.14
Doctor–patient communication capability (max 30 points)	27.00 (26.00, 28.00)	28.00 (27.00, 29.00)	Z = -3.685	0.000	0.90
Independent learning capability (max 30 points)	28.00 (27.00, 29.00)	27.00 (26.00, 28.00)	Z = -2.308	0.021	0.53

^a^Cohen’s effect size (d)

Teaching feedback from trainees is shown in Table 5. Note that the SDTM members enjoyed the training lesson process more than the trainees in the control group (4.28 versus 3.64; *P* < 0.001) with a large effect size (d = 0.96). However, the SDTM residents felt that more time should be set aside for each class compared to the control group (3.02 versus 3.54; *P* < 0.005) with a medium effect size (d = 0.75). This novel teaching method enabled the residents to show more initiative in class (4.41 versus 3.26; *P* < 0.001) with a large effect size (d = 1.82), and the effect of this teaching method on improving teacher-student interaction was not statistically significant (3.83 versus 3.77; *P* >0.05) with a small effect size (d = 0.08).

**Table 5:** Teaching feedback from trainees

	Control group	SDTM group	Statistics	*P* value	d^a^
The lessons were enjoyable	3.64 ± 0.707	4.28± 0.636	T =4.322	0.000	0.96
Time was tight	3.54 ± 0.682	3.02 ± 0.680	T = -3.369	0.001	0.75
Interaction between students and teacher was good	3.77 ± 0.842	3.83 ± 0.824	T = 0.346	0.887	0.08
I would act as a teacher	3.26 ± 0.637	4.41 ± 0.627	T = 8.171	0.000	1.82
I was able to learn a lot	3.69 ± 0.694	4.28 ± 0.708	T = 3.804	0.000	0.85

^a^Cohen’s effect size (d)

### Evaluation of long-term teaching effect

All the participants took the CMLE nine months after training. If the score falls below the pass mark (60 points), they fail. There were 74 (74/81) residents who had passed the clinical skill test of CMLE. As shown in Table 6**,** the results revealed that although the passing rate between the two groups showed no significant difference (*P* = 0.197), the item scores in the SDTM group were higher than those in the control group (*P* < 0.005). Cohen’s effect size suggested medium effect sizes practical significance (d = 0.2-0.8).

**Table 6:** Objective parameter comparison of the long-term teaching effect

	Control group	SDTM group	Statistics	*P* value	d^*^
Skill score of ^*^CMLE (max 100 points)	79.28 ± 9.714	83.43 ± 5.424	T = 2.103	0.039	0.47
Passing percentage of CMLE^a^	87.2% (34/39)	**95.2% (40/42)**	χ2 =1.663	0.197	0.29

*Cohen’s effect size (d); ^a^*CMLE* China medical licensing examination

## Discussion

In this study, we implemented the SDTM in clinical skill teaching for first-year standardized residency training residents in China. Compared to a conventional flipped learning approach that combined lecture with simulation, we confirmed that the SDTM significantly enhanced their performance of basic clinical skills in the following aspects: history-taking, long-term operating skill acquisition, and doctor–patient communication capability. However, the SDTM demonstrated no significant superiority in enhancing the short-term operating skill. The traditional method might be more efficient for promoting the independent learning capability of residents than that of the SDTM. We presumed that multiple factors might contribute to these results.

Observation and imitation are two crucial characteristics in medical skill education [18], and they have a variable influence on learning through format, content, and programming. The SDTM is derived from the terms and rules of the popular game called "spot the difference," which is a type of observational learning. Some studies have confirmed that game-based teaching methods can improve students' participation and subjective initiative [19], which is also proven in our study. There was some previous evidence supporting that observational learning can advance the development of clinical skills and motor learning in novices, especially in the context containing mixed factors [20, 21]. Nevertheless, those studies limited the training of students to only distinguishing different aspects, ignoring whether students know how to independently and correctly act after finding the faults. Timely correction and feedback after training were vital based on practice experience [22]. There is no emphasis on urgency during the observation period in previous studies, and in that case, the risk of learning burnout might increase. In our study, just as the game players were asked to find the differences between the two same scenes within the time allotted, the trainees needed to distinguish the flawed operations from the standard operations during the demonstration time. Several previous studies confirmed that the difference game promoted the establishment of multiple functions in the cerebrum cortex, including imitation, long-term memory, and concentration [4–9]. Few studies are devoted to whether this game-based teaching method could achieve better teaching results than the traditional teaching method [5–7, 9].

The SDTM is not a formalist and hidebound mode, and it can be designed as different assessment modes according to different training contents. The reference standards are essential elements for SDTM, and they can be included in flawless videos or represent a set of training criteria. We extended the two related approaches based on SDTM, which contained random differences and predesigned differences.

The CSTs involve a series of procedural and fine movements, a validated checklist with scoring items (Additional file 2). The DPs could be predesigned and inserted into the recording video according to complex or error-prone points in the checklist. This approach to design was more similar to the game experience, making better use of game-related promoting learning features. A recent study found that the observation of videos with the inclusion of errors did not improve skill learning. However, we believe this result may be related to evaluating only short-term outcomes without evaluating long-term outcomes [21]. In our study, although the short-term operating skill effect was actual, as reported in the literature [21], the long-term learning effect of SDTM was significantly superior to the control. This is consistent with literature that reports that games can promote long-term memory formation [8]. Moreover, the correction of movement in the inaccurate points of the demonstration in a timely manner is an essential step of training after observation, which has rarely been mentioned in the previous literature [18, 21, 22]. When they dictate and perform the corrections like a teacher, students can teach themselves by teaching others [23].

History taking is one of the primary clinical skills requiring more techniques and initiative than objective procedural operations [24]. To address these issues, we proposed that regardless of history-taking training in various scenes or cases, it should contain the essential content and a rational reasoning process (the checklist of Additional file 1), which were adopted as the reference standards of the DPs design. We believe that a complete medical history-taking content should include all the items in the checklist of Additional file 1. Since the DPs of medical history-taking are not aimed at a certain disease, the DP design should be based on holistic or subjective judgments. For example, the DPs for history-taking might include the following points: forget to ask about the predisposing factors, misjudge the concomitant symptoms, ignore negative symptoms inquiry and poor professional quality, and so on (Table 1). In the course of training, residents identified the DPs of their peers by comparing standard videos, so as to achieve the purpose of self-reflection and memory enhancement. The diversity of process presentation determines that the random DPs from trainees seem to be more appropriate for history-taking training and testing than predesign DPs. Our study confirmed that the SDTM combined with peer observation achieved both short-term and long-term teaching effects. To some extent, it might be more effective than the predesigned DP approach, which is consistent with recent evidence that supports the use of peer-assisted learning methods [16]. We presumed that integrating defect and advantage observations promotes introspection for mistakes and the acquisition of advantages, contributing to superior results. As the old Chinese proverb has it, there must be a teacher among any three fellows.

In addition to the objective evaluation of the teaching effect, this study also made a two-way subjective evaluation as the assessments of the teaching effect. These subjective evaluations from trainees reflected the recognition of the teaching method, as their satisfaction and engagement were the keys to achieving favorable program outcomes, which is in line with a previous study [16]. Teachers' subjective evaluation mainly came from their observation of students' clinical work. We found that comparing independent learning capability between the two groups showed a reverse trend to other evaluated items. We supposed that the DPs of the SDTM were designed aiming at exam-oriented education that might contribute to it, despite improving the efficiency of learning and score, which can result in learning burnout or stifling creativity or independent learning interest.

There were a few limitations, although we paid attention to the design of our study. One limitation is that although the students had received five basic clinical skills training sessions, we only conducted the assessments on two of them by random draw due to time and space limitations. Second, although the content of CMLE included all the items we had trained, our evaluations on long-term effects do not provide the information on which item of the CMLE results was most affected by SDTM. Because we could not obtain information on each score from the administrative department of health except aggregate scores of the clinical skills, further training and assessment for a single skill would have been necessary. Moreover, the training process of spotting the differences was comparing the former and the later operation video, which differed from the game. Therefore, if software engineers develop software that can simultaneously detect and flag differences in the two similar skill operating videos for clinical training, the SDTM might be more effective by taking the most advantages of this game and improving the efficiency of training. Finally, in further research, we would measure the learning effect at multiple time points so as to determine the time point for adding a booster, as the evidence of the retention effect would be an important feature in determining the utility of a training method.

## Conclusions

In conclusion, we have demonstrated the SDTM in primary clinical skill teaching for first-year residents in China. The teaching effect was achieved compared to using the traditional teaching method. Its teaching effect included not only the effect on short-term learning but also the long-term retention of learning outcomes. Although it may increase the risk of burnout, we believe the problem could be resolved by refining the design of diverse DPs and developing clinical teaching observational software. This study points to possibilities for optimizing the training curriculum in clinical skill training for novices by letting them observe both flawless and flaw demonstrations and then conduct corrective actions.

## Supplementary Information

**Additional file 1.**

**Additional file 2.**

## Data Availability

The raw data were deposited into the Mendeley Data dataset (https://data.mendeley.com/datasets) with DOI: https:// doi: 10.17632/dfrxj839p8.1.

## Abbreviations

FL: Flipped learning
CST: Clinical skills training
RPPC: Right posterior parietal cortex
SDTM: Spot the difference teaching method
DTE: Departmental rotation examination
PBL: Problem-based learning
CMLE: China medical licensing examination
PDF: Portable document format
DPs: Difference points
