# Supplementary material for: The application of the spot the difference teaching method in clinical skills training for residents
Source: BMC Med Educ. 2022 Jul 14;22:542. doi: 10.1186/s12909-022-03612-3 (PMC9281025; doi:10.1186/s12909-022-03612-3)
Supplement: Supplementary file 1 — Additional file 1. [file 12909_2022_3612_MOESM1_ESM.docx]

**History-taking evaluation scale**

| **Routine** | **Procedure** | **Value** | **Score** | **Remark** |
| --- | --- | --- | --- | --- |
| **History of present illness inquiry** | Predisposing factors: catch a cold, fatigue, diet, medicine, trauma, sentiments, etc. | **5** |  |  |
|  | The symptom features about the chief complaint | **10** |  |  |
|  | concomitant symptoms | **10** |  |  |
|  | Relevant negative symptoms | **10** |  |  |
|  | The past process of diagnosis and treatment includes inspection items, treatment methods, therapeutic effects, etc. | **10** |  |  |
|  | Inquire the general condition of patients like diet, sleep, urination and defecation, spirit and body weight changes since on onset. | **5** |  |  |
| **previous medical history inquiry** | The history of similar symptoms attacks or relevant diseases | **10** |  |  |
|  | Drug allergy history | **5** |  |  |
|  | Contagious disease history | **5** |  |  |
|  | The history of surgery | **5** |  |  |
|  | The history of any servere illness | **5** |  |  |
|  | Family history: family members healthy | **5** |  |  |
|  | Marital and Obstetrical history; menstrual history (female); feeding history(child); Bad habits | **5** |  |  |
| **Professional quality** | Reasoning ability; Focused inquiry | **10** |  |  |
| **Total points** |  | **100** |  |  |
