# Supplementary material for: The application of the spot the difference teaching method in clinical skills training for residents
Source: BMC Med Educ. 2022 Jul 14;22:542. doi: 10.1186/s12909-022-03612-3 (PMC9281025; doi:10.1186/s12909-022-03612-3)
Supplement: Supplementary file 2 — Additional file 2. [file 12909_2022_3612_MOESM2_ESM.docx]

**cardiopulmonary resuscitation evaluation scale**

| **Routine** | **Procedure** | **Value** | **Score** | **Remark** |
| --- | --- | --- | --- | --- |
| **Pre-resuscitation assessment** | Quickly position and correct consciousness judgment (tap patient’s shoulder) | **2** |  |  |
|  | Check pupils; pulse checking site (ipsilateral carotid artery) and pulse checking time (5–10s); breathing checking (looking, listening, and feeling) and pulse checking time (5–10s) | **5** |  |  |
|  | Call for help immediately | **2** |  |  |
|  | The patient is in the supine position; Place a hardboard under the patient's back | **3** |  |  |
| **CPR procedure** | Stand on the right side of the patient; Correct chest compression site (the junction of the middle and lower 1/3 of the sternum) | **10** |  |  |
|  | Shoulders right over the hands and straight elbows | **3** |  |  |
|  | Fingers extended and inter-sectioned; Fingers off the chest; The hands should never be away from the chest wall during compression | **10** |  |  |
|  | Correct compression depth (1.5–2 inches) | **5** |  |  |
|  | Correct compression rate (30 compressions was completed between 16 and 20s, which was approximately 100–120 compressions per minute) | **10** |  |  |
|  | Count aloud and chest wall fully recoil | **2** |  |  |
|  | Remove secretions and foreign bodies from mouth and nose; Keep the airway opening (head tilt and chin lift) | **5** |  |  |
|  | Open the mouth with one hand and pinch the nose with the other hand; Effective ventilation (visible chest wall movement); Each ventilation lasts 1s | **10** |  |  |
|  | Observe chest expansion and loosen the nose before the next. | **5** |  |  |
|  | Ratio of compression / ventilation (30:2) | **10** |  |  |
|  | Judge the resuscitation effect after 5 cycles and CPR-free interval less than 15 s (between each circle including pulse re-checking) | **10** |  |  |
| **Professional quality** | Swift action, fluent operation and communication skills | **8** |  |  |
| **Total points** |  | **100** |  |  |

**dressing change evaluation scale**

| **Routine** | **Procedure** | **Value** | **Score** | **Remark** |
| --- | --- | --- | --- | --- |
| **Preparation** | Wear a hat and mask. | **5** |  |  |
|  | Properly expose the scope of surgical incision. | **5** |  |  |
|  | Wash hands(dictating). | **2.5** |  |  |
|  | Preparing items: two dressing bowls, two tweezers, iodophor cotton balls and dressings | **5** |  |  |
|  | Patient informed consent | **2.5** |  |  |
| **the process of dressing change** | Remove the outer dressing by hand and place the inner layer of the dressing upwards. | **10** |  |  |
|  | Remove the inner dressing with a pair of tweezers. | **5** |  |  |
|  | The removed dressings should be placed in a dressing bowl containing dirt. | **5** |  |  |
|  | A pair of tweezers contacts the incision, and the other are used to transfer the cleaning items of the dressing bowl. | **10** |  |  |
|  | Keep the tip of the tweezers lower than the holding end. | **5** |  |  |
|  | Observe and examine the wound. | **5** |  |  |
|  | Take the incision as the center and disinfect it from inside to outside twice (the infected wound is disinfected from outside to inside). | **5** |  |  |
|  | The disinfection area is 3-5 cm around the incision；the second disinfection scope should be narrower than the first. | **5** |  |  |
|  | Apply sterile gauze with reasonable layers, and the edge of the gauze should exceed the incision by 3cm. | **5** |  |  |
|  | The direction of the adhesive tape should be perpendicular to the long axis of the torso. | **5** |  |  |
|  | The changed dressing should be discarded in the medical garbage bag. | **5** |  |  |
|  | Inform the patient of relevant precautions after dressing change. | **5** |  |  |
| **Professional quality** | Aseptic technique, fluent operation and patient communication | **10** |  |  |
| **Total points** |  | **100** |  |  |

# disinfection and surgical drapes placement evaluation scale (neck)

| **Routine** | **Procedure** | **Value** | **Score** | **Remark** |
| --- | --- | --- | --- | --- |
| **Preparation** | Wash hands(dictating); wear a hat and mask. | **5** |  |  |
|  | Properly expose the scope of skin disinfection. | **5** |  |  |
| **Disinfection of operation area** | Hold a bowl containing iodophor cotton balls in one hand and a forceps in the other; The surgeon stands on the patient's right side. | **5** |  |  |
|  | Disinfection range: Up to the lower lip, down to the nipple line, both sides to the anterior edge of the trapezius muscle. | **15** |  |  |
|  | Take the incision as the center and disinfect from inside to outside (2 to 3 times); there is no blank space in the disinfected area. | **10** |  |  |
|  | The scope of each disinfection should not exceed the previous one. | **5** |  |  |
|  | Keep the tip of the sterilizing forceps lower than the holding end. | **10** |  |  |
| **Drape the surgical area** | Four sterile towels, partially folded, are used to cover around the proposed incision, with the folded part facing down and close to the incision. The skin exposure of the operation field after placing surgical drapes should not be too broad. | **10** |  |  |
|  | Place four towels in the correct order: the lower part, opposite side, head side and trainee side; Four towel clamps fixed the drapes, the fixed method is correct. | **15** |  |  |
|  | Lay two more oversized drapes on the upper and lower parts of the incision. | **5** |  |  |
|  | Lay the third layer of surgical hole drape, align the hole with the proposed incision, cover the anesthesia frame at the foot end of the drape, and hang both sides and the head end more than 30cm the edge of the operating table. | **5** |  |  |
| **Professional quality** | Aseptic technique and fluent operation | **10** |  |  |
| **Total points** |  | **100** |  |  |

**wearing and taking off the operating gown and sterile gloves evaluation scale**

| **Routine** | **Procedure** | **Value** | **Score** | **Remark** |
| --- | --- | --- | --- | --- |
| **Wearing operating gown** | Pick up the folded operating gown and keep hands away from the lower one. | **5** |  |  |
|  | Lift both ends of the collar of the operating gown with both hands and unfold it, with the side of the belts facing outwards | **10** |  |  |
|  | Throw up the gown slightly, put the hands forward and up and insert them into the sleeves simultaneously. Extend the hands forward (not over the shoulders). The assistant shall help put on the operating gown from the back so that the hands stretch out the cuffs. | **10** |  |  |
|  | Lean forward slightly so that the belts hang away from the operating gown. | **5** |  |  |
|  | Hold the midpoints of the left and right belts with crossed hands and pass them to the back. The assistant should catch them and tie a knot from the back. | **10** |  |  |
|  | The hands and forearms should not be above the shoulders and below the waist. | **5** |  |  |
| **Wearing sterile gloves** | Hold the folded parts of the gloves with the left hand and take them out of the glove bag; Insert the right hand into the right glove. | **10** |  |  |
|  | Insert the four fingers of the gloved hand (except the thumb) into the folding part of the left glove; insert the left hand into the glove; turn the folded part of the gloves over the cuff of the operating gown. | **10** |  |  |
| **taking off operating gown and sterile gloves** | Ask the assistant to untie the knots on the back and neck | **5** |  |  |
|  | Ask the assistant to hold the collar of the surgical gown, and pull it down and forward, so that the glove cuff could turn over on the wrist. | **10** |  |  |
|  | Insert one hand into the folded part of the other glove and pull off the glove; hold the other glove's inner surface with the hand that has taken off the glove, and pull off the second glove. | **10** |  |  |
| **Professional quality** | During the removal of gloves, the hands shall not touch the outside of gloves. | **10** |  |  |
| **Total points** |  | **100** |  |  |
